# Chronic Lymphocytic Leukemia Presenting as a Subcortical Watershed Infarct

**DOI:** 10.1155/2019/2089359

**Published:** 2019-01-09

**Authors:** Mridul Gupta, Divita Singh, Patrick Lee, Sandhya Kadiyam

**Affiliations:** ^1^Internal Medicine, Monmouth Medical Center, Long Branch, New Jersey, USA; ^2^Ambulatory Care Pharmacy Resident, University of Florida, Gainesville, Florida, USA; ^3^Hematology and Oncology, Monmouth Medical Center, Long Branch, New Jersey, USA

## Abstract

Internal watershed infarcts (WI) involve white matter between deep and superficial arterial systems of middle cerebral artery. These infarcts are considered to be either from low blood flow or microembolism. Anemia is an extremely rare cause of watershed infarcts. Very few cases of hemolytic anemia causing watershed cerebral infarcts have been reported. Chronic lymphocytic leukemia (CLL) is frequently complicated with secondary autoimmune cytopenia such as autoimmune hemolytic anemia (AIHA), immune thrombocytopenia (ITP), and pure red cell aplasia. AIHA is present in about 7–10% of patients with CLL. AIHA from CLL presenting as WI is an extremely rare phenomenon with no previously published case reports to the best of our knowledge.

## 1. Introduction

Chronic lymphocytic lymphoma (CLL) is a chronic lymphoproliferative disorder characterized by progressive monoclonal accumulation of functionally incompetent lymphocytes. It is the most common leukemia in the Western world and accounts for about 25–30% of all leukemia cases in the Unites States [1]. Autoimmune cytopenias are a relatively common hematologic complication and can be refractory to treatment often requiring prolonged steroid use in combination with second-line therapies and transfusion of blood products. Systemic hemodynamic compromise arising from autoimmune hemolytic anemia (AIHA) in CLL is a rare occurrence. We hereby present a case of cortical watershed infarct (WI) arising from AIHA which in turn was found to be the presenting manifestation of CLL.

## 2. Case Presentation

A 72-year-old man with a history of hypertension and cerebrovascular accident (CVA) 20 years ago without significant residual weakness presented to the emergency department with a 3-day history of right-sided facial droop, slurred speech, and clumsiness of the right hand. He had a 20 pack-year smoking history. His family history revealed a father who died suddenly at the age of 47 from an unknown cause, a brother with acute leukemia, and a sister with myocardial infarction at the age of 37 years.

On examination, he had mild right facial droop, mild dysarthria, right pronator drift, and 4/5 motor strength in the right upper and lower extremity. The remainder of the neurological examination was unremarkable. Other significant findings in physical examination were mild hepatosplenomegaly with axillary and cervical lymphadenopathy. Hematological investigations on admission showed severe anemia with a hemoglobin (Hb) level of 44 g/L, leukocytes 42.8 × 10^9^/L, lymphocytes 35.95 × 10^9^/L, neutrophils 4.28 × 10^9^/L, and a platelet count of 120 × 10^9^/L. Further investigations were most consistent with AIHA with an unconjugated hyperbilirubinemia (2.7 mg/dL), elevated LDH (444 IU/L), low haptoglobulin (<15 mg/dL), an elevated reticulocyte count 83.62 × 10^9^/L (7.4%), and a positive direct antiglobulin test (DAT) with both IgG and anti-C3d.

Peripheral blood flow cytometry showed a monoclonal B-cell population with surface lambda-positive population and positive for CD 45, CD 19, CD 20 (weak), CD 22, CD 23, CD 5, and CD 38 (partial). This immunophenotype pattern was consistent with diagnosis of CLL. The clinical picture was consistent with modified Rai stage III CLL. The patient had multiple autoantibodies on cross match.

**Figure 1:**
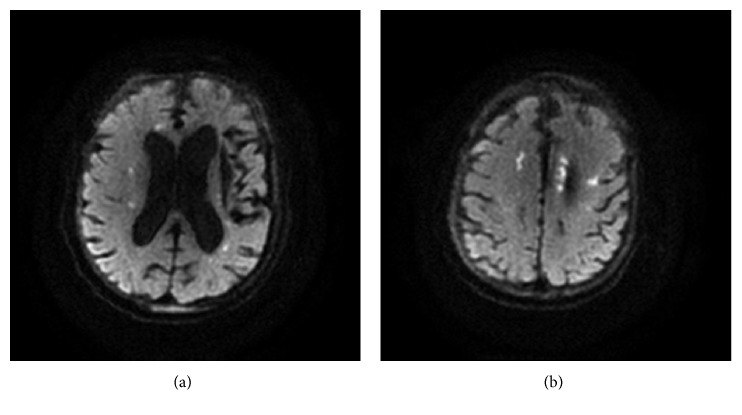
Brain MRI showing small areas of diffusion restriction with T2 hyperintensity over (a) anterior horn of right lateral ventricle and (b) posterior horn of left lateral ventricle and left corona radiata, consistent with acute infarcts.

Computed tomography (CT) of the head was negative for acute bleed or mass effect but was suspicious for evolving stroke. It also showed an area of encephalomalacia in the left basal ganglia related to an old infarct or hemorrhage. The patient could not receive thrombolytic therapy due to severe anemia. Brain magnetic resonance imaging (MRI) showed multiple small areas of diffusion restriction with corresponding mild T2 hypersensitivity in the bilateral corona radiata and centrum semiovale. These findings were consistent with subcortical WI in a characteristic “string of pearls” pattern (Figure 1). Magnetic resonance angiogram (MRA) of the head and neck was negative for stenosis in any of the major vessels. Transthoracic echocardiogram showed normal left and right ventricle systolic function with normal ejection fraction. Subsequent transesophageal echocardiogram did not show any left atrial thrombus or atrial mass. Interatrial septum was also intact. No arrhythmias were noted on telemetry during the admission; however, on a subsequent admission the patient was noted to have paroxysmal atrial fibrillation.

The patient was transfused to a goal Hb of 8 g/dL with least incompatible packed red cell (PRBC) transfusions. A total of 7 units of PRBC were transfused during the hospital stay, which the patient tolerated without any significant reactions. AIHA was treated with intravenous immunoglobulin (IVIG) 1 g/kg body weight daily for five days followed by 1 mg/kg of prednisone daily. At the time of discharge, the patient's Hb was 8.8 g/dL and LDH had normalized. There was no significant change in the platelet count with steroids. At discharge, oral prednisone was continued for four months and gradually tapered off over the next two months.

His blood counts stabilized with steroids and IVIG. About 1 year after initial presentation, he was found to have worsening diffuse adenopathy in the posterior cervical and axillary areas. PET/CT done to evaluate the possibility of Richter's transformation showed extensive bulky hypermetabolic lymphadenopathy in the head and neck region, bilateral axilla, bilateral hila, mediastinum, and throughout the mesentery and retroperitoneal distribution of the abdomen and pelvis. Maximum standardized uptake value (SUV) of 4.64 was noted in the right posterior cervical region. At this time, the patient's LDH was 538 IU/L. Repeat flow cytometry showed a monoclonal surface lambda population which was positive for CD 20 (weak), CD 19, CD 5, CD 23, CD45, and CD 22. This pattern was again consistent with CLL.

The patient's clinical status declined rapidly. Plan for excision lymph node biopsy to rule out Richter's phenomenon had to be deferred due to severe debilitation and refractory AIHA. He was started on obinutuzumab with chlorambucil but had an anaphylactic reaction to obinutuzumab which led to discontinuation after 2 doses. His diffuse adenopathy did respond to the two doses of obinutuzumab with near-complete resolution over the next several days. The patient however remained very debilitated and confined to bed and further consideration of therapy was not appropriate and further staging of his lymphoma was deferred until his condition improved. His hospital course was complicated with recurrence of autoimmune hemolytic anemia, which was managed with blood product transfusions, steroids, and IVIG as before. He was eventually started on rituximab for refractory AIHA. He was in the middle of his second course of rituximab before his counts began to improve.

## 3. Discussion

CLL is the most common leukemia in the Western countries. In the United States, CLL accounts for approximately 25 to 30% of all leukemia cases [1]. It is more common in the elderly with a median age of 71 years at diagnosis [2]. There is also male predominance with a ratio of 1 : 6: 1 approximately [1]. CLL is frequently complicated with secondary autoimmune cytopenias such as AIHA, immune thrombocytopenia (ITP), pure red cell aplasia (PRCA), and autoimmune agranulocytosis (AG). Incidence of these cytopenias is estimated to be approximately 4.3–9.7% in patients with CLL in most studies [3]. However, as these cytopenias often precede CLL diagnosis or occur at any time during the disease process, these numbers are likely to be an underestimation [4]. Systemic hemodynamic compromise arising from AIHA in CLL is an extremely rare occurrence. Our patient presented with a cortical WI arising from AIHA which in turn was found to be the presenting manifestation of CLL.

WI or border zone infarcts are ischemic lesions in the brain parenchyma located at the junction of territories supplied by 2 different non-anastomosing arterial systems. Two main types of WI described in literature are the cortical WI and the internal WI. The cortical WI occurs between cortical territories of anterior cerebral artery (ACA), middle cerebral artery (MCA), and posterior cerebral artery (PCA). The internal WI affects corona radiata between territories supplied by superficial and deep perforators of MCA and/or centrum semiovale between territories of superficial perforators of ACA and MCA [5].

WI represents about 10% of all brain infarcts in autopsy studies [6]. Actual incidence may be much higher since these infarcts are usually mild, asymptomatic, and nonfatal. In a study by Sorgun et al. to determine the etiologic subtypes of WI, out of 32 patients with a subcortical watershed infarct, 21 patients had large-artery atherosclerosis, 5 patients had cardio-embolism, and 3 had stroke due to causes such as aneurysm, hypercoagulability from chronic myeloid leukemia, and vasculitis [7]. Cortical WI are commonly related to microembolic phenomenon, while internal WI occur as a consequence of hemodynamic impairment [8].

Radiologically, internal WI can be either confluent or partial. Confluent infarcts are larger cigar shaped along the lateral ventricle. Partial internal WI can be either in the form of isolated single lesions or a chain-like “rosary” pattern. This rosary-like pattern in the centrum semiovale strongly supports the hemodynamic mechanism theory of causing WI [5]. Partial internal WI can sometimes be difficult to differentiate from leukoaraiosis and might even coexist in elderly patients.

Atrial fibrillation and atheromatous plaques are major offenders in causing microembolic diseases. Hemodynamic compromise leading to WI can arise from severe stenosis or occlusion of craniocervical arteries and/or systemic hypoperfusion in the setting of cardiac arrest or systemic hypotension. Hypoperfusion can further potentiate effects by affecting the clearance of microemboli [9]. Systemic hypotension and carotid disease are major etiologic causations of hemodynamic compromise leading to WI. Hemodynamic compromise from severe anemia leading to cerebral hypoperfusion and subsequent WI is an extremely rare phenomenon. Our patient presented with severe hemolytic anemia which was later found to be the presenting manifestation of CLL.

AIHA occurs in about 7% of patients with CLL and an additional 7–14% of patients may have a positive DAT without clinical evidence of hemolysis. [3] These numbers are variable based on the sample population and duration of follow-up. Moreno et al. [10] analyzed data from 960 Spanish CLL patients over 29 years and reported autoimmune cytopenias in 7.29% (70) patients out of which 5.1% (49) had AIHA, 2.08% (20) had ITP, and 0.10% (1) had Evan's syndrome. Out of these patients with CLL, 19 were found to have autoimmune cytopenias at diagnosis, 3 prior to diagnosis, and 48 during the course of the disease. In an observational study of 1750 CLL patients with a 10-year follow-up at Mayo Clinic, autoimmune cytopenias were diagnosed in 4.3% patients, with 2.3% patients developing AIHA and 2% developing ITP [11]. Data from 964 patients from an Israel CLL study group followed for 35 years revealed 11 (1.14%) cases with AIHA at diagnosis, 55 (5.70%) with DAT positivity without AIHA at diagnosis, and 43 (4.46%) developed AIHA during follow-up [12].

Various theories have been postulated to explain the pathophysiology behind these secondary cytopenias. Nonmalignant B cells in CLL can produce high affinity immunoglobulin G (IgG) directed against the antigens on erythrocytes and platelets, leading to AIHA and ITP [13]. CLL cells can also act as efficient antigen presenting cells, leading to T-cell response with subsequent autoimmunity [13]. Cytokines production from CLL cells can result in inhibition of normal erythropoiesis or megakaryopoiesis, leading to development of AIHA or ITP [14]. CLL cells can rarely act as effector cells, secreting a pathological monoclonal antibody [15]. CLL cells may also be stimulated through their polyreactive B-cell receptor (BCR) that recognizes autoantigens [15].

Although DAT is the most important diagnostic test, AIHA is also known to occur with a negative DAT. DAT-negative AIHA can arise from either lower affinity antibodies, antibodies in concentrations below threshold for the test, or autoantibodies of IgA class which are not detected with the current testing methods [14].

Warm AIHA is caused by warm agglutinins which are predominantly IgG antibodies that react with protein antigens on the red blood cell (RBC) surface at body temperature and cause extravascular hemolysis. Cold agglutinins cause cold AIHA and are predominantly IgM antibodies that generally react with polysaccharide antigens on the RBC surface only at temperatures below that of the core temperature of the body and commonly cause intravascular hemolysis. Differentiating between these two forms is important in terms of diagnosis and treatment.

Initial management of patients presenting with acute anemia consists of supportive care and may require transfusion of blood products based on local protocols that consider the blood count and patient symptoms. When treatment of CLL is not indicated, immunosuppression can be used alone. Immunosuppression with systemic glucocorticoids is used as a first-line treatment for AIHA from warm agglutinins. Prednisone 1 mg/kg is given for 4 weeks, which can be tapered over 1-2 months. Use of intravenous immunoglobulin (1 g/kg body weight daily for 5 days) in the beginning can also be considered. Chemotherapeutic agents are not to be used as first-line agents for CLL associated with AIHA or ITP [16]. Steroids are less effective for AIHA with cold agglutinins and are not considered as first-line treatment [14]. Instead rituximab is the preferred first-line agent for AIHA patients with cold antibodies [17].

If underlying CLL also needs to be treated, steroids can be used to manage AIHA while chemotherapeutic agents are being used. Rituximab also has some activity against CLL, so it can be used in monotherapy or in combination with other chemotherapy agents [18]. Rituximab has been used successfully in patients with warm AIHA in combination with or without steroids as first-line treatment or as a second-line agent following failure from first-line management. Recent consensus guidelines from an international workshop on CLL recommend against using targeted agents such as rituximab as first-line agents before steroids in the treatment of AIHA in CLL patients [16].

For patients who fail to respond to first-line therapy, cyclophosphamide, azathioprine, cyclosporine, and mycophenolate mofetil have been used as second-line agents in the treatment of DAT-positive AIHA [16]. The role of splenectomy in AIHA associated with CLL is not clearly defined. In a study by Akpek et al. in patients with AIHA in CLL, splenectomy resulted in partial response in half of the patients and no response in the other half [19]. More recently, Hill et al. concluded that laparoscopic splenectomy was safe and effective in treating AIHA associated with CLL in patients who had failed medical therapy [20]. Patient preference had often elevated use of rituximab prior to splenectomy.

Patients not responding to first- or second-line treatment within 4–6 weeks must be considered for alternate treatment options such as cyclophosphamide, mycophenolate mofetil, cyclosporine, or azathioprine [4]. Alternate causes of anemia should always be ruled out in refractory and relapsed cases prior to escalating treatment.

Rituximab has also been used in combination with other cytotoxic agents to provide more “CLL-directed treatment.” Combination therapies with rituximab for autoimmune cytopenias include rituximab–cyclophosphamide–dexamethasone (RCD) [21, 22], rituximab–cyclophosphamide–vincristine–prednisone (R-CVP) [23], and rituximab–bendamustine [24]. Evidence for these combination modalities is limited to small case series.

Recently, newer monoclonal antibodies have been studied for their role in the treatment of refractory cases of AIHA with CLL. Nader et al. reported a case of rituximab-refractory AIHA associated with CLL which was successfully treated with ofatumumab [25]. Ibrutinib is a bruton tyrosine kinase inhibitor that has been approved for the initial treatment or in relapsed/refractory CLL cases. Furthermore, in patients with del(17p)/TP53 mutations, ibrutinib is the preferred choice for initial therapy. Although initial clinical trials of ibrutinib had excluded patients with uncontrolled hemolysis, recently there have been case reports of ibrutinib successfully being used for CLL-associated AIHA either as monotherapy or in combination with rituximab/steroids [26, 27].

In a randomized controlled trial from a German CLL study group, obinituzumab combined with chlorambucil was found to be superior to rituximab–chlorambucil combination or chlorambucil monotherapy in previously untreated patients [28]. Of note, in this trial, patients with AIHA were not considered suitable for enrollment.

Our patient was given a trial of obinituzumab–chlorambucil based on its superiority over the rituximab–chlorambucil combination. Despite favorable response in his lymph nodes after two treatments, it was not reinitiated due to severe reaction and near-complete remission of his adenopathy.
